# Contribution of Environmental Surveillance Toward Interruption of Poliovirus Transmission in Nigeria, 2012–2015

**DOI:** 10.1093/infdis/jiv767

**Published:** 2016-04-02

**Authors:** Ticha Johnson Muluh, Abdullahi Walla Hamisu, Kehinde Craig, Pascal Mkanda, Etsano Andrew, Johnson Adeniji, Adefunke Akande, Audu Musa, Isiaka Ayodeji, Gumede Nicksy, Richard Banda, Sisay G. Tegegne, Peter Nsubuga, Ajiboye Oyetunji, Ousmane Diop, Rui G. Vaz, Ado J. G. Muhammad

**Affiliations:** 1 World Health Organization, Country Representative Office, Abuja, Nigeria; 2 World Health Organization, Regional Office for Africa, Brazzaville, Congo; 3 National Primary Health Care Development Agency, Abuja; 4 University of Ibadan, Nigeria; 5 Global Public Health Solutions, Atlanta, Georgia; 6 World Health organization, Head Quarters, Geneva, Switzerland

**Keywords:** environmental surveillance, poliovirus, sewage, wild poliovirus, circulating vaccine derived poliovirus

## Abstract

***Background.*** Cases of paralysis caused by poliovirus have decreased by >99% since the 1988 World Health Assembly's resolution to eradicate polio. The World Health Organization identified environmental surveillance (ES) of poliovirus in the poliomyelitis eradication strategic plan as an activity that can complement acute flaccid paralysis (AFP) surveillance. This article summarizes key public health interventions that followed the isolation of polioviruses from ES between 2012 and 2015.

***Methods.*** The grap method was used to collect 1.75 L of raw flowing sewage every 2–4 weeks. Once collected, samples were shipped at 4°C to a polio laboratory for concentration. ES data were then used to guide program implementation.

***Results.*** From 2012 to 2015, ES reported 97 circulating vaccine-derived polioviruses (cVDPV2) and 14 wild polioviruses. In 2014 alone, 54 cVDPV type 2 cases and 1 WPV type 1 case were reported. In Sokoto State, 58 cases of AFP were found from a search of 9426 households. A total of 2 252 059 inactivated polio vaccine and 2 460 124 oral polio vaccine doses were administered to children aged <5 year in Borno and Yobe states.

***Conclusions.*** This article is among the first from Africa that relates ES findings to key public health interventions (mass immunization campaigns, inactivated polio vaccine introduction, and strengthening of AFP surveillance) that have contributed to the interruption of poliovirus transmission in Nigeria.

Cases of paralysis caused by poliovirus have decreased by >99% since the World Health Assembly's resolution to eradicate polio in 1988 [1]. Substantive progress has been made toward this goal, but further work is required [2]. The World Health Organization (WHO) strategy for monitoring wild polioviruses (WPVs) and mutated vaccine polioviruses, also known as “circulating vaccine-derived polioviruses” (cVDPVs), is to identify virus isolates from AFP cases and contacts [3, 4]. The absence of paralytic cases of poliomyelitis is an unreliable marker of global polio eradication, and it must be accompanied by biological monitoring, especially in high-risk areas [4]. Vaccine virus can survive in sewage treatment plants and in the environment for several months [4]. It is essential to identify WPV and VDPV in clinical and environmental samples, to measure the effectiveness of polio eradication strategies [4].

Environmental surveillance (ES) for poliovirus is of growing importance as the eradication target is approached [5]. Because poliovirus is shed from infected subjects with and those without paralysis, ES is thought to allow sampling of the entire population [6]. ES continues to play an important role in the eradication of wild poliovirus (WPV) from the remaining polio-endemic countries of Pakistan and Afghanistan. Several ES studies have been performed but have shown very limited linkage to public health interventions, especially in Africa. T. Hovi et al reported that ES findings in Egypt in 2000 resulted in intensified immunization campaigns and improved AFP surveillance throughout Egypt [7]. This article seeks to showcase the linkage of ES to key public health interventions that contributed positively to the interruption of poliovirus transmission in Nigeria.

## METHODS

Nigeria commenced ES in 2011 and expanded it in phases. The criteria used for the prioritization of states to start ES were based on the risk of persistent WPV circulation (despite good surveillance performance) that include noncompliance to immunization activities (supplementary immunization activities and routine immunization), proximity to polio-endemic states, as well as the capacity of the single polio laboratory (having limited capacity) to handle samples. Based on the above criteria, 11 high-risk states were selected for initiation and expansion of ES. However, because of the need to gain experience before scaling up, pilot ES was initiated in Kano in 2011 (3 sites), and in 2012 this was expanded to Sokoto at 4 sites and Lagos with 5 sites. In 2013, ES was initiated in Kaduna (3 sites), Abuja (Federal Capital Territory [FCT]; 2 sites), and Borno (4 sites). Subsequently, 4 other states were brought on board in 2014, Kebbi (3 sites), Katsina (3 sites), Jigawa (3 sites), and Yobe (3 sites). In 2015, ES was initiated in Adamawa State at 3 sites. Thus, by May 2015, 11 states were participating in ES, using 38 sewage-collecting sites. The selection of states and sites was preceded by capacity building by various stakeholders involved in the process, particularly personnel in the Federal Ministry of Health, the National Primary Health Care Development Agency, and state ministries of environment and health.

### Sampling Sites

Sampling sites were selected along open drainage canals in the cities, owing to the absence of modern sewage draining systems in the country, except in one site in FCT, where a modern sewage treatment system exists. Site selection was carefully done as per World Health Organization standard guidelines [8]. Care was taken to avoid areas that could contain chemical waste from industries.

### Sampling Technique and Schedule

The grap method was used, whereby 1.75 L of midstream raw flowing sewage were collected at the time of maximum flow (generally, 6 am–8 am, to avoid heat), using stainless iron buckets. Collected samples were maintained at about 4°C until reaching the laboratory in Ibadan. The frequency of collection was once every 4 weeks at each site except in Sokoto and Kaduna (these are states where WPV type 1 [WPV1] and circulating VDPV type 2 [cVDPV2] have recently been isolated from the environment) where sewage water was collected every fortnight. Samples were collected by trained staff from the state ministries of environment and were supervised by the disease surveillance and notification officers in the local government areas and WHO personnel, using an in-built questionnaire on smart phones. The collector shipped the samples to the Ibadan polio laboratory, using reverse cold chain via public transport. The temperature of the sample was recorded upon arrival in the laboratory, and then the sample was processed for polioviruses typing.

### Laboratory Methods

The 2-phased separation method was used for sewage sample concentration with quality control ensured by the regional reference laboratory same as it is done for the characterizing of polioviruses obtained from AFP cases [8].

ES data were processed into the polio laboratory database by the Ibadan polio laboratory, which also doubles as a testing site for AFP samples. Data sets were shared with the federal government and the WHO in a similar manner as for the existing AFP surveillance system. From the WHO, feedback on ES data was shared with stakeholders, including the national polio emergency operations center (EOC), weekly, but there was immediate notification of polioviruses (WPVs or cVDPVs) that were identified. Laboratory data quality control was discussed quarterly during data harmonization meetings organized at the national level.

The findings reported from ES were disseminated during the meeting of the expert review committee (ERC) on polio eradication and routine immunization, which directs the national EOC in Abuja to provide response interventions to limit the spread of polioviruses in high-risk states in the country.

## RESULTS

**Figure 1.**
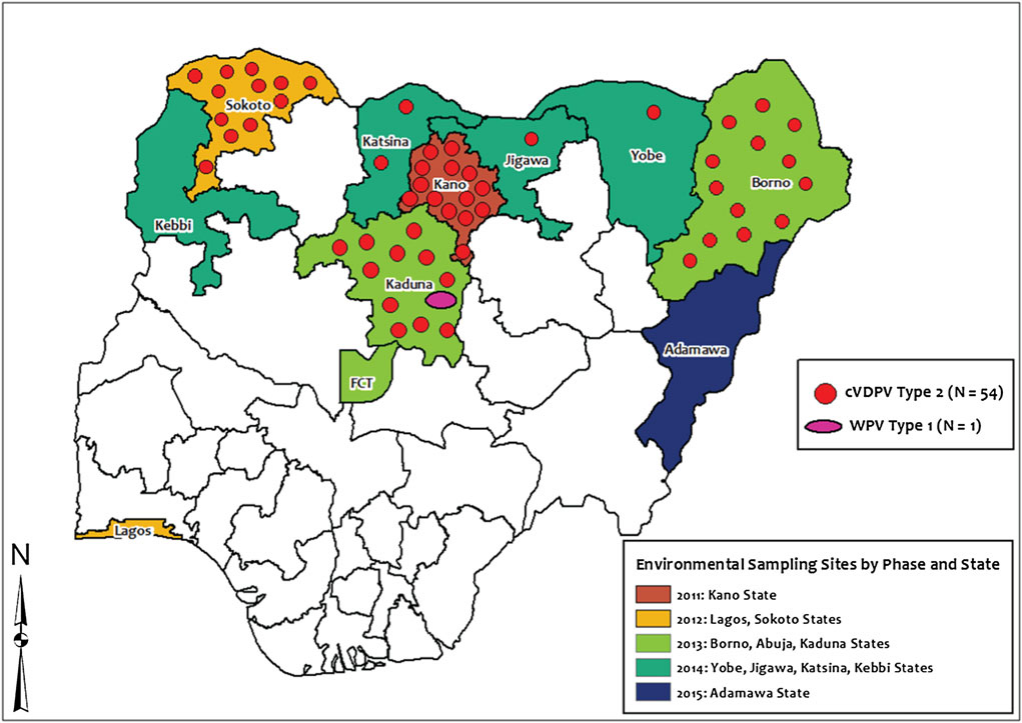
Map of Nigeria, showing phased introduction of environmental surveillance in the states and the number of polioviruses isolated in 2014. Abbreviations: cVDPV, circulating vaccine-derived poliovirus; WPV, wild poliovirus.

Ninety-seven cVDPV2 cases, 14 WPV1 cases, and 1 WPV3 case were reported from ES in Nigeria between 2012 and 2015 (January–June). In 2012, 1 WPV3 case was reported at the Makoko site in Lagos State. In 2014, 54 cVDPV2 cases were reported from Borno (13 cases), Yobe (1 case), Katsina (2 cases), Jigawa (1 case), Sokoto (12 cases), Kano (14 cases) and Kaduna (11 cases) (Figure 1).

From April 2014 to June 2015, children were given 81 million oral polio vaccine (OPV) doses in response to the isolation of polioviruses by ES in Kaduna State (Table 1). One WPV was reported in Kamacha Tudun Bridge ES site in April 2014. ES conducted from 4 June to 2 August 2014 reported 7 cVDPV2 cases from 4 sites located in Rigasa, Lemanchi Kaura Junction, and Kamacha Tudun Bridge. ES samples collected from sites in Kamacha Tudun Bridge in August 2014, October 2014, and March 2015 were positive for cVDPV2 (Table 1).

**Table 1.** Types of Polioviruses Reported From Environmental Sites and Number of Children Immunized in Response Activities, Kaduna State From April 2014 to June 2015

Sewage Collection Site	Date of Sewage Collection	Poliovirus Isolated	Vaccine Used	Doses, No.
Kamacha Tudun Bridge	5 Apr 2014	WPV1	bOPV	10 380 174
Rigasa, Lemanchi Kaura Junction, Kamacha Tudun Bridge	4 Jun–2 Aug 2014	7 cVDPV2	tOPV	40 959 929
Kamacha Tudun Bridge	9 Aug 2014	cVDPV2	tOPV	7 397 543
Kamacha Tudun Bridge	7 Oct 2014	cVDPV2	tOPV	8 976 539
Kamacha Tudun Bridge	23 Oct 2014	cVDPV2	tOPV	7 292 348
Kamacha Tudun Bridge	4 Mar 2015	cVDPV2	bOPV	6 465 432
Total	…	…	…	81 471 965

Abbreviations: bOPV, bivalent oral polio vaccine; cVDPV2, circulating vaccine-derived poliovirus type 2; tOPV, trivalent oral polio vaccine; WPV1, wild poliovirus type 1.

In response to ES results, 9527 households were surveyed in 102 settlements during a retroactive case search for AFP cases conducted by 68 trained searchers. All 53 AFP cases found were old cases, and all had been reported by the existing AFP surveillance system, except for one that was missed in Tudun Wada A ward (Table 2).

**Table 2.** Finding From Retroactive Case Search in Sokoto Upon Isolation of an Ambiguous Vaccine-Derived Poliovirus in February 2015

Ward	Searchers Trained, No.	Settlements Visited, No.	Households Visited, No.	AFP Cases Found, No.		No Unreported AFP Cases
				Overall	Date of Onset >60 d Earlier	
Tudun Wada B	16	19	2824	17	17	0
Tudun Wada A	20	28	2270	15	15	1
Rijiya A	8	16	1260	3	3	0
Gagi A	24	39	3173	18	18	0
Total	68	102	9527	53	53	1

Abbreviation: AFP, acute flaccid paralysis.

In Borno and Yobe states, both inactivated polio vaccine (IPV) and OPV were given to children aged <5 years through 25 accessible wards (85%) and 17 accessible wards (95%), respectively, in response to ES results. In Borno State, 85% of wards were accessible for delivery of 1 371 493 IPV doses and 1 477 920 OPV doses to children aged <5 years through 2214 health camps (Table 3).

**Table 3.** Receipt of Inactivated Polio Vaccine (IPV) and Oral Polio Vaccine (OPV) Among Children Aged <5 Years at Health Camps by Wards in Borno and Yobe States in June 2014

State	LGAs, No.	Percentage of Wards Accessible	Health Camps, No.	Children Who Received IPV, No.	Children Who Received OPV, No.
Borno	25	85	2214	1 371 493	1 477 920
Yobe	17	95	1925	880 566	982 204
Total	42	90	4139	2 252 059	2 460 124

Abbreviation: LGA, local government area.

In Yobe State, similar to what happened in Borno State, 95% of wards were accessible and were used to administer 880 566 IPV doses and 982 204 OPV doses to children aged <5 years through 1925 health camps.

## DISCUSSION

We found that ES findings were used in Nigeria to guide programmatic public health responses to polioviruses by the national polio EOC as per standard operating procedures in the polio outbreak response guidelines, outlined in the 2015 National Polio Emergency Plan of Action. These response interventions in Kaduna, Borno, Yobe, and Lagos contributed to interruption of poliovirus transmission. This similarly happened in Egypt between January and December 2001, when 8 OPV immunization campaigns in children aged <5 years were used to interrupt WPV transmission as a result of poliovirus reported from ES [9].

We also found that ES findings triggered the accelerated introduction of IPV in Borno and Yobe states in June 2014. Both states are located in northeast Nigeria, which is facing security challenges. The accessible wards were used to provide IPV/OPV, to rapidly build herd immunity. Similar to this approach in Nigeria, Pellegrinelli et al stated in 2013 that the epidemiological and virological surveillance of the environment, even in polio-free countries, enables early detection of any reemergence of WPV or cVDPV strains, with different implications for public health measures [10]. In Finland during the 1984–1985 outbreak of polio, WPV3 was revealed by ES in several provinces without the occurrence of paralytic cases [9].

In Nigeria, the impact of IPV introduction in these 2 states (Borno and Yobe) was felt because by June 2015 no polioviruses (WPV or cVDPV) had been identified by the ES and AFP surveillance systems since June 2014. A study in Yogyakarta Province, Indonesia, during 2007 revealed that immunity induced by IPV introduction was sufficiently robust to prevent the emergence and circulation of VDPVs [1]. After IPV introduction in Borno and Yobe states, its introduction into the routine immunization program is being scaled up nationwide.

We also found out that, in Sokoto State, ES findings triggered the conduct of a retroactive case search in high-risk communities that drained into the canal that reported an ambiguous VDPV2 isolate (ie, an isolate whose ultimate source is unknown). During this house-to-house active case search, education materials were used by trained searchers to sensitize high-risk communities on reporting of AFP cases. This describes another dimension of public health interventions triggered by ES findings that helped to strengthen the AFP reporting system.

We have not exhaustively described all the public health interventions that took place in Nigeria in response to ES findings from 2012 to 2015 because we set out to showcase major interventions performed because of evidence provided by ES findings that contributed to the interruption of poliovirus transmission in Nigeria.

Although the findings of this article are limited to the high-risk states in Nigeria, it is also true that intense poliovirus transmission was generally limited to the same states between 2012 and 2015, the period under analysis in this article. These focused interventions contributed toward the interruption of poliovirus transmission locally, with an impact felt nationwide.

In conclusion, experience from Nigeria confirms that ES can detect the introduction and silent circulation of WPV and cVDPV [3]. Between 2012 and 2015, Nigeria made timely use of information from ES to trigger public health interventions that contributed to the progress made toward the interruption of poliovirus transmission. The last WPV1 from the AFP surveillance was reported in July 2014 from Kano, and by the end of November 2015, no polio case had since been reported in the country. However, ES is still restricted in the high-risk states for poliovirus; its expansion to other states will be guided by viral epidemiology, laboratory capacity to cope with workload, and financial support. We recommend that ES, which is currently being scaled up in Nigeria to monitor the Polio Strategic Endgame Plan (2013–2018) [11], should link findings to strategies to improve surveillance and to support the implementation of rigorous outbreak responses when positive samples are found [12].
